# Evaluation of an Early-Warning System for Heat Wave-Related Mortality in Europe: Implications for Sub-seasonal to Seasonal Forecasting and Climate Services

**DOI:** 10.3390/ijerph13020206

**Published:** 2016-02-06

**Authors:** Rachel Lowe, Markel García-Díez, Joan Ballester, James Creswick, Jean-Marie Robine, François R. Herrmann, Xavier Rodó

**Affiliations:** 1 Catalan Institute of Climate Sciences (IC3), Carrer Doctor Trueta, 203, 3a, 08005 Barcelona, Spain; markel.garcia@ic3.cat (M.G.-D); joan.ballester@ic3.cat (J.B.); xavier.rodo@ic3.cat (X.R.); 2 World Health Organization (WHO) Regional Office for Europe, European Centre for Environment and Health, Platz der Vereinten Nationen 1, 53113 Bonn, Germany; creswickj@who.int; 3 National Institute of Health and Medical Research, INSERM U988 and U1198, University of Montpelier, Building 24, Place Eugène Bataillon-CC105, 34095 Montpellier, Cedex 05, France; jean-marie.robine@inserm.fr; 4 Ecole Pratique des Hautes Etudes, 75014 Paris, France; 5 Division of Geriatrics, Department of Internal Medicine, Rehabilitation and Geriatrics, Geneva University Hospitals, University of Geneva, Chemin du., Pont-Bochet, 1226 Thônex, Switzerland; francois.herrmann@hcuge.ch; 6 Catalan Institution for Research and Advanced Studies (ICREA), Passeig de Lluís Companys, 23, 08010 Barcelona, Spain

**Keywords:** temperature, extremes, heat wave, mortality model, early warning system, climate services

## Abstract

Heat waves have been responsible for more fatalities in Europe over the past decades than any other extreme weather event. However, temperature-related illnesses and deaths are largely preventable. Reliable sub-seasonal-to-seasonal (S2S) climate forecasts of extreme temperatures could allow for better short-to-medium-term resource management within heat-health action plans, to protect vulnerable populations and ensure access to preventive measures well in advance. The objective of this study is to assess the extent to which S2S climate forecasts could be incorporated into heat-health action plans, to support timely public health decision-making ahead of imminent heat wave events in Europe. Forecasts of apparent temperature at different lead times (e.g., 1 day, 4 days, 8 days, up to 3 months) were used in a mortality model to produce probabilistic mortality forecasts up to several months ahead of the 2003 heat wave event in Europe. Results were compared to mortality predictions, inferred using observed apparent temperature data in the mortality model. In general, we found a decreasing transition in skill between excellent predictions when using observed temperature, to predictions with no skill when using forecast temperature with lead times greater than one week. However, even at lead-times up to three months, there were some regions in Spain and the United Kingdom where excess mortality was detected with some certainty. This suggests that in some areas of Europe, there is potential for S2S climate forecasts to be incorporated in localised heat–health action plans. In general, these results show that the performance of this climate service framework is not limited by the mortality model itself, but rather by the predictability of the climate variables, at S2S time scales, over Europe.

## 1. Introduction

Temperature-related illness and death is a continuing public health concern, and in many regions is presenting an increasing burden on public health systems due to climate change [1,2]. The Intergovernmental Panel on Climate Change (IPCC) concluded that there is medium confidence that the observed warming in the climate system has increased heat-related human mortality in some regions [3], and heat waves have been responsible for more fatalities in Europe over the past decades than any other extreme weather event [4].

Europe emerges as an especially responsive area to temperature rise under climate change, particularly during the warm season [5]. The anomalous and persistent heat of the summer of 2003 caused over 70,000 additional deaths across twelve countries in Western Europe [6]. This event revealed the lack of reactivity of society and health services to such an extreme event. Since then, some European countries have implemented strict measures and protocols to minimize the negative effects of heat waves. Some of these efforts have successfully reduced the impact of the following extreme events, such as in France, where mortality rates were reduced by 70% lower than expected during the 2006 episode with regard to the expected mortality [7]. Certain adverse health effects could easily be avoided if informed decisions were made prior to heat waves, to protect vulnerable populations, such as children and the elderly, and ensure access to preventive measures well in advance [8,9].

A WHO study [10] estimated an increase in annual heat-related deaths of 15,280 additional deaths in 2030, and 32,152 additional deaths in 2050, for the over 65 year-old population in the WHO European Region (including central Asia), assuming no adaptation. However, this increase might be offset by a similar reduction in cold-related human mortality before 2050, whereas such compensation would not take place afterwards, with a resulting average of 15,000 excess deaths per year [11]. Moreover, heat waves are relatively infrequent compared with the total number of hot days, thus the effect of heat waves is likely to represent only a fraction of the total heat impact [12]. Deaths attributable to extreme heat are roughly as frequent as those attributable to moderate heat [13]. The effects of exposure can be directly heat-related (heat stroke, heat fatigue and dehydration) or heat stress, which can contribute to a worsening of respiratory and cardiovascular diseases, electrolyte disorders and kidney problems [8]. A myriad of other indirect health effects should also be considered, including food shortages and malnutrition, social conflicts and aggravation of chronic illnesses.

Temperature-related illnesses and deaths are largely preventable [7,14,15]. One of the most effective health system preparations for this emergency is the development and implementation of action plans for preparedness and response, such as heat-health action plans (HHAP). Development, implementation and subsequent evaluation and improvement of these preparedness and response plans would lead to a reduction in temperature-related mortality and stronger climate-resilient health systems. A HHAP relies on early-warning systems for timely activation and to allow for longer-term resource planning. Forecast data and projections for temperature are primarily used by health systems within the decision-making frameworks of these plans. Improved climate forecasts would allow for better short-to-medium-term resource management within health systems and would help authorities prepare and respond ahead of heat waves.

HHAPs can be evaluated based on inclusion of nine core elements [16,17,18]. These elements focus on short-term activation and implementation during the event, defining specific roles and actors. Three of these core elements are of specific interest for the development of climate services for temperature and health: a timely alert system, preparedness of the health/social care system, and long-term urban planning. Due to their speed of implementation, these three core elements have potentially different lead-times for forecasting: alert systems triggering plan activation are typically days in advance. However, if a country had not experienced such an extreme temperature event for several years, earlier warning could provide sufficient time to review and update existing action plans. Longer-term health system preparedness and better resource management would benefit from sub-seasonal-to-seasonal (S2S) forecasts to allow for increasing capacity in the health system and improving the resilience of the primary health services, especially in the case of prolonged and/or recurrent heat waves where mortality significantly increases with intensity and duration of heat waves. Long-term urban planning for extreme weather occurs on annual-to-decadal (or even longer timescales) and includes cross-sectoral issues such as increasing green and blue spaces, changes in building design and improvement in housing stock, changes in land-use decisions, reductions in energy consumption, and improved transport policies.

In an assessment of the 18 countries of the WHO European Region with known heat–health action plans, 16 had a clearly defined alert system and a health system preparedness component, all included an information plan, but only four included long-term urban planning within the HHAP itself [16]. None of these HHAPs routinely incorporated S2S climate forecasts. Thus, there exists potential for these forecasts to be incorporated into pre-existing plans in the European Region, whilst countries yet to develop a HHAP could incorporate such information at the design stage.

In a previous study [19], the performance of a climate-driven mortality model to provide probabilistic predictions of exceeding emergency mortality thresholds for a heat wave and a cold spell scenario was evaluated. The mortality model was formulated using observed (reanalysis) apparent temperature data. This observed temperature data was then used to produce spatio-temporal probabilistic mortality estimates for a heat wave and cold spell scenario. The model showed considerable skill, particularly for the heat wave scenario (1–15 August 2003), successfully anticipating the occurrence or non-occurrence of mortality rates exceeding the emergency threshold (75th percentile of the mortality distribution) for 89% of the 54 regions, given a probability decision threshold of 70%. The use of observed apparent temperature in the mortality model represented an upper bound to forecast skill, given a perfect climate forecast. In this study, we replace the observed apparent temperature with forecast apparent temperature at 8 different lead times, from 1 day to 3 months, to drive the model and produce probabilistic mortality forecasts ahead of the 2003 heat wave event in Europe. This allows the assessment of the extent to which S2S climate forecasts could be incorporated into HHAP, to support timely public health decision-making ahead of imminent extreme temperature events in Europe.

## 2. Experimental Section

### 2.1. Temperature-Mortality Model

Daily mortality data corresponding to 187 NUTS2 regions across 16 countries in Europe were obtained from 1998 to 2003 (see [6] for details). The NUTS2 regions (*i.e.*, second level of the Nomenclature of Territorial Units for Statistics) are basic regions of the economic territory of the European Union for the application of regional policies. Due to the extremely large differences in population between these regions (from 18 million in North Rhine-Westphalia to less than 100,000 in some Slovenian regions), regions were grouped in 54 larger and more homogeneously populated areas. Several factors were taken into account in this process, such as geographical proximity, state borders (e.g., similar warning systems or adaptation measures might be applied in each country), and similarities in regional temperature/mortality dependencies or comfort temperatures [11]. Location-specific average mortality rates, at given temperature intervals over the entire time period, were modelled to account for the increased mortality observed during both high and low temperature extremes and differing comfort temperatures between the 54 aggregations [11,19]. The temperature-mortality dependency for each aggregation was estimated as follows:

Apparent temperature, defined by the following equation, is the climatological input to the mortality model:

(1) $$T_{app}=-2.653+0.994T_{air}+0.0153T_{dewpt}^{2}$$

where *T_app_* is the apparent temperature, *T_air_* the air temperature and *T_dewpt_* the dew point temperatures (at 2 metres), in degrees Celsius, from the reanalysis ERA-Interim dataset [20]. We refer to this reanalysis data as “observed” apparent temperature data herein. To relate the climate variables to mortality data for the 54 aggregated regions, the apparent temperature data at gridpoints found inside the region were identified and averaged for each time step. In case the region was smaller than the gridsquares, the value of the nearest neighbour grid to the centroid of the region was used.

The range of apparent temperatures was divided in equally spaced intervals. Days belonging to each interval were grouped, and daily temperature and mortality data within each interval were averaged. Interval mean mortality was smoothed using a centred 31-term filter, corresponding to nearly 3 °C intervals. The lowest value defines the interval of comfort temperature. This threshold divides the range of temperatures into “warm” and “cold” tails [11]. The model used to fit the temperature-mortality curves was formulated as follows:

(2) $$y_{ik}∼N(\alpha_{j}+\beta_{1j}x_{ik}+\beta_{2j}{x^{2}}_{ik}+\beta_{3j}{x^{3}}_{ik},\sigma_{j}^{2})$$

where *y_ik_* is the logarithm of the average mortality rate (per million population) in region *i,* and at temperature interval *k*. Then, for each region *i*, the log mortality rate was formulated as a non-linear function of temperature, *x_ik_*, (a third order polynomial) with location specific intercept *α_j_*. This formulation directly corresponds to the linear predictor of a Poisson count model, with the population in each region as a model offset. When comparing observed to predicted mortality in the subsequent analysis, the log mortality incidence rates are transformed to mortality counts. Note, parameters are fitted separately for the warm tail (*j* = *w*) and cold tail (*j* = *c*), depending on whether the temperature is greater than (*x_ik_* ≥ *x_im_*) or less than (*x_ik_* < *x_im_*) the comfort temperature (*i.e.*, the temperature of minimum mortality), *x_im_*. The comfort temperature occurs twice a year around June and September, defining a summer season of warm tail temperatures with non-linear mortality sensitivity and a long three-season period (autumn, winter and spring) of cold tail temperatures with near-linear mortality increases with decreasing temperature [11]. This model framework distinguishes between warm and cold periods, without restricting the model to calendar months, thus allowing temperature extremes, which might occur outside the typical range of “warm” or “cold” months, to be detected.

The model was fitted in a Bayesian probabilistic framework, with 1000 samples of mortality rates generated for each time step [19]. This allows the simulation of probabilistic predictions of daily mortality in space and time, given any user-defined emergency and probability decision thresholds. In order to simulate mortality predictions for a heat wave scenario, spatio-temporal observed apparent temperature data, *x_it_*, where *t* is the time step (daily), are combined with 1000 samples of the parameters estimated from the warm tail model (*j* = *w*):

(3) $$y_{it}∼N(\alpha_{w}+\beta_{1w}x_{it}+\beta_{2w}{x^{2}}_{it}+\beta_{3w}{x^{3}}_{it},\sigma_{w}^{2}),\;\mathrm{if}\;x_{it}\geq x_{im}$$

This gives 1000 samples of daily mortality rates for each region and each day, *y_it_*. Daily mortality rates can then be averaged for the climatological events of interest (e.g., the heat wave in August 2003).

### 2.2. Climate Forecasts

Climate forecasts from two different datasets were used: The European Centre for Medium-Range Weather Forecasts (ECMWF) seasonal forecast system version 4 (System4) and the ECMWF sub-seasonal forecast system. Hindcasts (*i.e.*, retrospective forecasts) for the 2003 event were obtained using the most recent state-of-the-art forecast system.

Seasonal forecasts seek to take advantage of aspects of the climate system with long-term memory, such as the oceans, to predict climate anomalies one or more months ahead of a given season. To estimate uncertainty, each forecast consists of an ensemble of forecasts, obtained by perturbing the initial conditions. Therefore, the information produced can be summarised in probabilistic terms. The ECMWF System4 [21] consists of a global coupled ocean-atmosphere model initiated from reanalysis. The available hindcasts cover 30 years (1981–2010). In the present study two seasonal forecasts, with 15 ensemble members each, were used, one starting 1 July 2003 (one month before the event), the other starting 1 May 2003 (three months before the event).

Sub-seasonal forecasts (or extended-range forecasts) lie between medium-range weather forecasts and seasonal forecasts, and take advantage of the predictability of phenomena like the Madden-Julian oscillation or jet stream blocking events [22]. The climate model used for the sub-seasonal forecasts is similar to the model used to produce seasonal forecasts, but with a higher resolution. The model is run twice a week, out to 46 days ahead. The available hindcasts cover 20 years (1995–2015), with 10 ensemble members. In the present study, 6 sub-seasonal forecasts were used with the following start dates: 30-07-2003, 27-07-2003, 23-07-2003, 20-07-2003, 16-07-2003 and 13-07-2003, which correspond to lead times of 1, 4, 8, 11, 15 and 18 days, respectively.

Both sets of forecasts are produced using multiple climate model runs, which produce high frequency (up to hourly) data. The difference between sub-seasonal and seasonal forecasts is the complexity of the model and the lead time: the longer the lead time, the longer the period over which variables should be averaged, in order to find predictability. However, forecast providers usually supply data for every day in the forecast period, to enable the study of indicators, such as the number of heat wave days in a given month or season. In our case, the mortality model needs daily frequency data as input. For this study, daily mortality forecasts are aggregated over a longer period of 15 days (see below).

As with the observed temperature data, both sub-seasonal and seasonal forecasts were aggregated to the 54 regions, as described above. Therefore, 54 time series with a daily resolution, for each ensemble member and forecast lead time, were computed. Both series for 2-metre temperature and dew point temperature were generated and then combined, following Equation (1), to produce regional sub-seasonal and seasonal forecasts of apparent temperature.

The regional apparent temperature forecasts were then bias-corrected, following [23], before using them as an input to the mortality model. This technique removes “model drift” [24] and aligns forecasts with past observations. Both sub-seasonal and seasonal apparent temperature forecast ensembles data were then used to run the mortality model, generating 1000 samples of daily mortality rates for each region, each day and each ensemble member (*i.e.*, 10,000 samples for the sub-seasonal forecasts and 15,000 samples for the seasonal forecasts, for each day and region). By incorporating the whole ensemble of temperature forecasts in the probabilistic Bayesian framework, the additional source of uncertainty generated by using forecast instead of observed temperatures is accounted for, as well as uncertainty in the mortality estimates themselves.

For comparison, observed apparent temperature (from ERA-Interim reanalysis) was used to run the mortality model, producing 1000 samples of daily mortality rates for each region and each day. This allows an assessment of skill loss when replacing observed with forecast climate data to run the mortality model. As a case study, daily mortality rate samples were averaged for the heat wave period of interest, 1–15 August 2003, using both observed and S2S forecast climate data as inputs to the mortality model.

Following [19], region specific emergency thresholds of mortality rates were set at the 75th percentile (3rd quartile) of the mortality distribution, for the days in the time period in which temperatures were greater than the comfort temperature, *i.e.*, in the warm tail distribution. This allowed us to assess the ability of the temperature-driven mortality model to determine mortality rates exceeding this emergency threshold. The probability of exceeding this threshold in each region was determined using the mortality model driven by (i) observed apparent temperature data (*i.e.*, the proportion of 1000 samples that exceeded the location specific emergency threshold); (ii) the sub-seasonal apparent temperature forecast data, with lead times of 1, 4, 8, 11, 15 and 18 days (*i.e.*, the proportion of 10,000 samples that exceeded the location specific emergency threshold) and (iii) the seasonal apparent temperature forecast data, with lead times of 1 and 3 months (*i.e.*, the proportion of 15,000 samples that exceeded the location specific emergency threshold).

## 3. Results

### 3.1. Excess Mortality Probability Maps Using Climate Forecasts at Increasing Lead Times

**Figure 1:**
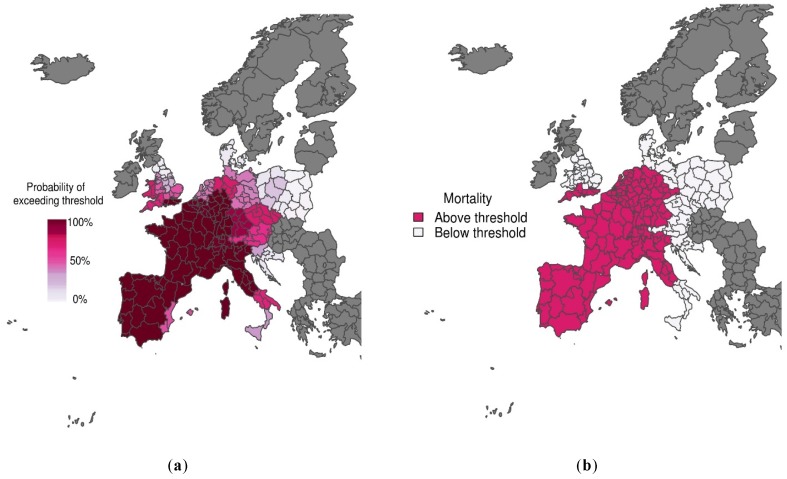
(**a**) Probabilistic map of exceeding emergency daily mortality threshold (75th percentile of daily mortality distribution in the warm tail) using a mortality model driven with observed apparent temperature data during the heat wave scenario (1*–*15 August 2003); (**b**) Corresponding observations for the same period. The graduated colour bar represents the probability of exceeding the mortality threshold (ranging from 0%, pale colours, to 100%, deep colours). Source: [19].

**Figure 2:**
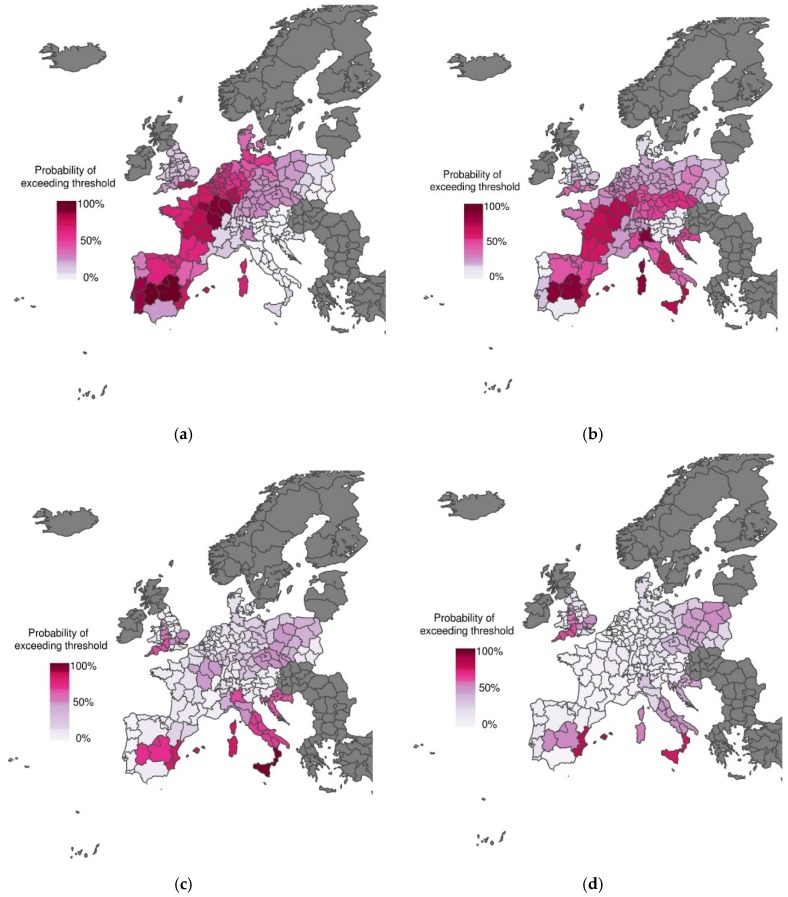

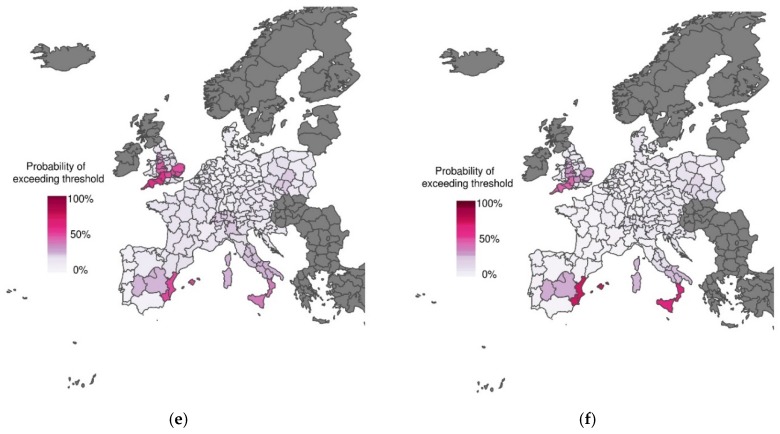
Probabilistic maps of exceeding emergency daily mortality threshold (75th percentile of daily mortality distribution in the warm tail) during the heat wave scenario (1–15 August 2003) using sub-seasonal forecasts of apparent temperature at lead times of (**a**) 1 day; (**b**) 4 days; (**c**) 8 days; (**d**) 11 days; (**e**) 15 days; (**f**) 18 days. The graduated colour bar represents the probability of exceeding the mortality threshold (ranging from 0%, pale colours, to 100%, deep colours).

**Figure 3:**
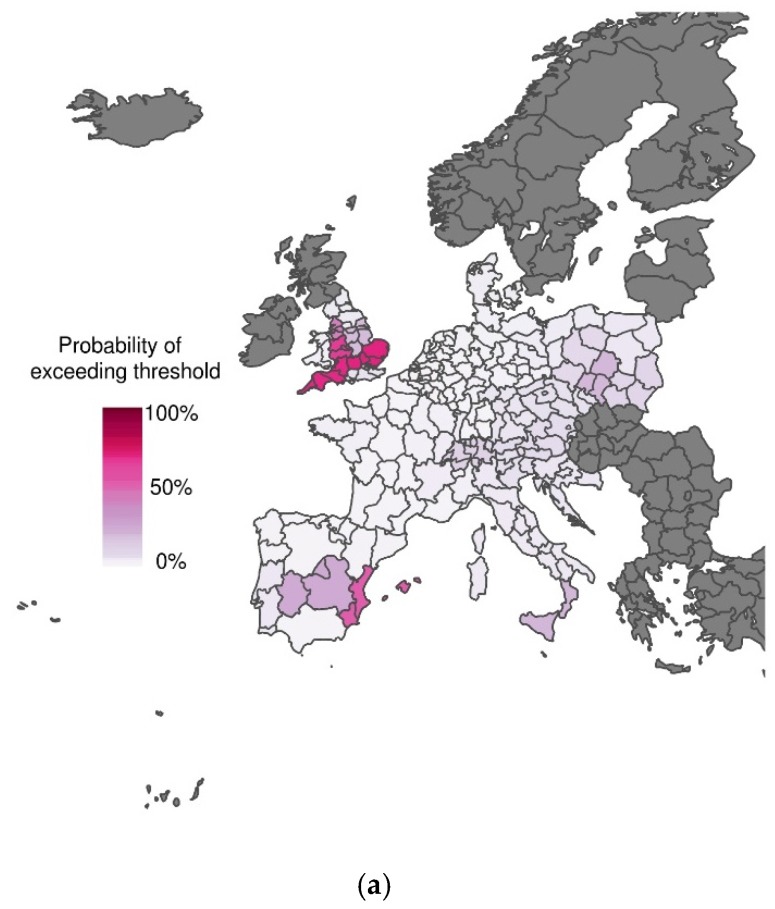

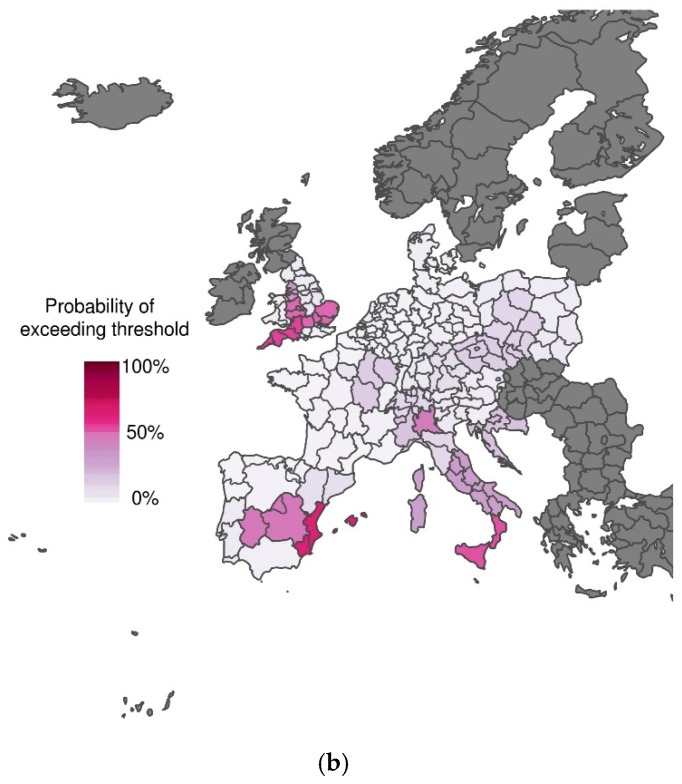
Probabilistic maps of exceeding emergency daily mortality threshold (75th percentile of daily mortality distribution in the warm tail) during the heat wave scenario (1–15 August 2003) using seasonal forecast apparent temperature at lead times of (a) 1 month and (b) 3 months. The graduated colour bar represents the probability of exceeding the mortality threshold (ranging from 0%, pale colours, to 100%, deep colours).

Figure 1a shows the predicted probability of mortality rates exceeding the 75th percentile of the mortality distribution for the heat wave period 1–15 August 2003. The corresponding observations (*i.e*., whether the mortality rate exceeded the threshold or not) are displayed in Figure 1b. There is considerable agreement between predictions and observations, with the model correctly predicting with high confidence that mortality would exceed the emergency threshold across most of Spain, France and Northern Italy (see [19] for further details). We use this example of a mortality prediction, using observed temperature, as a reference (or benchmark). This allows us to compare mortality predictions, using forecasts of apparent temperature at increasing lead times from sub-seasonal (1 to 18 days, see Figure 2) to seasonal (1 and 3 months, see Figure 3) time scales.

At lead times of 1 and 4 days, excess mortality is reasonably well predicted, with high certainty of exceeding the emergency threshold for much of western Europe (compare Figure 2a,b to Figure 1b). As lead time increases, the mortality forecasts become less certain and the sub-seasonal climate forecasts begin to erroneously detect excess mortality in southern Italy (compare Figure 2c–f) with Figure 1b). Interestingly, even at seasonal time scales, excess mortality is correctly predicted with some degree of certainty for some regions, including central and eastern Spain and southwestern United Kingdom (compare Figure 3a,b to Figure 1b). These results are promising for the development of more localised early warning systems.

**Figure 4:**
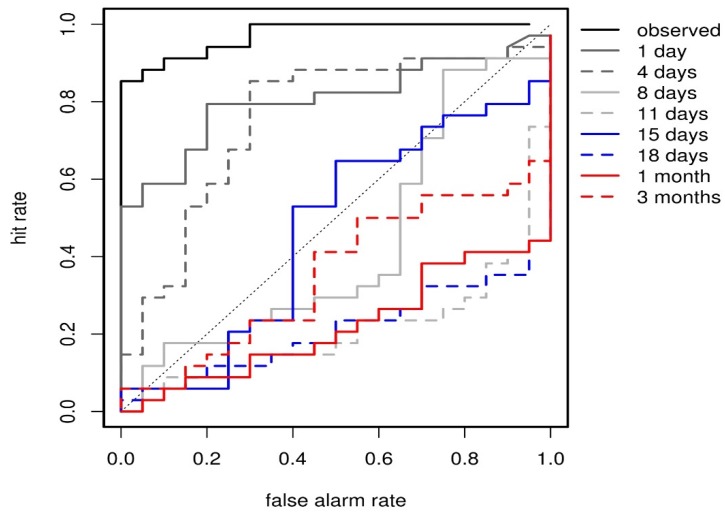
ROC curves for the binary event of exceeding the emergency mortality threshold of the 75th percentile in each of the 54 regions for the heat wave scenario (1*–*15 August 2003), using the probabilistic mortality model driven by forecast apparent temperature data at lead times ranging from 1 day, 4 days, 8 days, 11 days, 15 days, 18 days, 1 month and 3 months. The ROC curve for the mortality model driven by observed apparent temperature data is shown for reference (black curve).

### 3.2. Skill Assessment for Increasing Forecast Lead Time

Relative operating characteristic (ROC) curve can be used to indicate the hit rates and false alarm rates that would result from using different probability decision thresholds to determine a binary event (in this case, exceeding or not the emergency mortality threshold in regions across Europe). The ROC score or AUC (equivalent to the Area Under the modelled ROC curves), is a widely used measure of skill [25]. The ROC score characterises the quality of a forecast system by describing the system’s ability to anticipate correctly the occurrence or non-occurrence of pre-defined events. A ROC score value of 0.5 indicates zero skill while a value of 1 repr in this case exceeding esents perfect skill.

**Figure 5:**
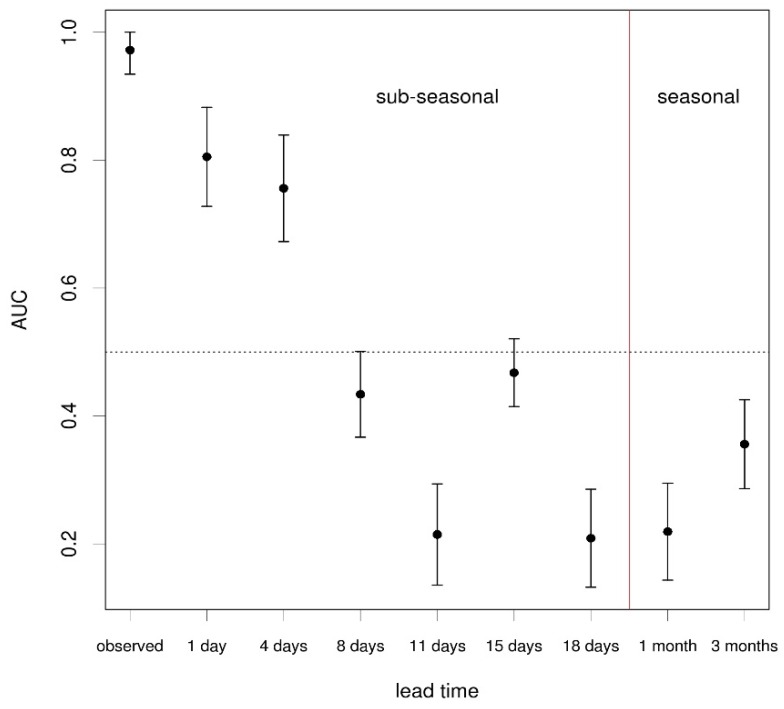
The ROC score or area under the ROC curve (AUC) and associated 95% confidence intervals for the binary event of exceeding the emergency mortality threshold of the 75th percentile in each of the 54 regions for the heat wave scenario (1*–*15 August 2003), using the probabilistic mortality model driven by forecast apparent temperature data at lead times ranging from 1 day, 4 days, 8 days, 11 days, 15 days, 18 days (sub-seasonal forecasts), 1 month and 3 months (seasonal forecasts). The AUC for the mortality model driven by observed apparent temperature data is shown for reference. The dotted horizontal line indicates the skill threshold of AUC = 0.5. The red vertical line distinguishes lead-times at sub-seasonal (less than a month) and seasonal (greater than a month) time scales. Results are also displayed in the accompanying table.

**Table:** 

**Climate Forecast Lead Time**	**Mean AUC (95% Confidence Intervals)**
Observed	0.97 (0.93, 1.00)
1 day	0.81 (0.73, 0.88)
4 days	0.76 (0.67, 0.84)
8 days	0.43 (0.37, 0.5)
11 days	0.21 (0.14, 0.29)
15 days	0.47 (0.41, 0.52)
18 days	0.21 (0.13, 0.29)
1 month	0.22 (0.14, 0.29)
3 months	0.36 (0.29, 0.43)

Figure 4 shows ROC curves for the probabilistic mortality predictions for the 2003 heat wave scenario, using forecast temperatures at increasing lead times, ranging from 1 day to 3 months. The ROC curve obtained using the observed temperature data is also shown. Figure 5 shows the corresponding ROC score or AUC (*i.e.*, the area under the modelled ROC curve) for each lead time, and associated confidence intervals, computed with 2000 stratified bootstrap replicates [26]. Results show that significant overall skill exists for the two shorter lead times (1 and 4 days). This is an important finding, since the non-linear nature of the mortality model makes it very sensitive to errors in temperature, especially in the warm tail. These mortality forecasts are more informative than simple temperature forecasts because they use empirical temperature-mortality relationships that take into account the different degrees of adaptation of the regions to warm temperatures. AUC for lead times longer than 4 days are below the reference value of 0.5. In the case of the ROC score, this implies that the forecast is no better than the mortality prediction derived from a climatological or random climate forecast. The “negative” skill found using longer lead times is the result of the climate forecasts not being able to reproduce the record-breaking summer heat wave, and instead, simulating a local warm event in southern Italy. Therefore, excess mortality is incorrectly predicted in southern Italy, leading to potential false alarms.

## 4. Discussion 

In this study, we have used S2S climate forecast data at different lead times (*i.e.*, 1, 4, 8, 11, 15 and 18 days, and 1 and 3 months) to drive a mortality model [11,19], and compared results to the predictions inferred from observational climate data [19]. Results have shown that there is a smooth and monotonically decreasing transition in skill between excellent predictions with observational data and predictions with almost no skill a week in advance and beyond. These results show that the performance of the scheme is not limited by the mortality model itself, but is instead constrained by the skill of the climate forecasting system. However, even at longer lead times, the system is able to forecast excess mortality with some confidence in certain regions of Spain (see Figure 2 and Figure 3). These regions coincide with the regions were skill is found in the seasonal forecast of summer months [21]. While this may be a coincidence, results suggest that forecasts with longer lead times are possible in these regions. This needs to be confirmed in further studies, using temperature and mortality data over a longer time period.

This simplistic mortality model is formulated using only one thermal variable. Other socio-economic factors, which can strongly influence mortality rates during heat waves, are not explicitly considered in the model. However, the temperature-mortality relationships and the emergency thresholds are region dependent. Therefore, they implicitly include different sensitivity to temperatures in each region. Despite its simplicity, results show that the model successfully predicts excess mortality for the 2003 heat wave event when observed temperature data is used. Predictability is only lost when replacing observed with forecast temperature. Thus, the lack of skill at increasing lead times is due to the quality of the climate forecasts, rather than the temperature-mortality model itself.

This work indicates that the predictability of temperature-related mortality is strictly determined by the chaotic nature of the atmosphere. Indeed, temperature-related mortality is predictable as far as climate forecasts provide useful skill for the prediction of temperature variables. Beyond this window of around one week, the limited skill in climate forecasts prevents the accurate prediction of excess mortality at lead times up to a season ahead. Therefore, it is the lack of climate forecast skill in Europe that limits the predictability of temperature-related mortality for the majority of regions. However, there appears to be some predictability of temperature-related mortality in parts of Spain and the United Kingdom, even at lead times of 3 months. These areas deserve further attention to investigate the potential for more localised early-warning systems.

The spatial coverage of the mortality dataset allowed a Europe-wide assessment of the capability of state-of-the-art seasonal climate forecast to predict excess mortality in the region. However, this study is restricted by the relatively short time period for which the mortality data was available (1998–2003), thus providing only one example of an extreme heat wave for analysis. As more mortality data becomes available, an assessment of the loss of skill in predicting excess mortality with increasing forecast lead-time, for other more recent European heat waves, can be compared to corroborate the findings presented here.

Despite the limitations of this study, the results have strong implications for current research initiatives in Europe. As it is widely known, the Horizon 2020 programme of the European Commission is highlighting the importance of climate services for Europe and globally within the framework of European research and excellence. Particularly, its specific challenge is the provision of trustworthy science-based climate information to government, public and private decision-makers as a fundamental prerequisite for both properly managing the risks society is facing and seizing the opportunities this implies. However, if climate information is to improve the decision-making process and the resilience of society in Europe, climate services need to be based on skillful climate forecasts that can provide useful information as input data for the impact models. In that regard, our work highlights the current potential weaknesses of the climate service strategy for Europe, given that seasonal forecasts in this region are limited for informing certain impact models. Therefore, if climate services initiatives are to succeed in achieving their challenging goals, efforts should in parallel be directed towards the improvement of climate forecasting in this region of the planet. The limited skill of seasonal forecasts in Europe is linked to the low predictability of the planetary waves developing in the polar jet stream. However, several encouraging results have been published pointing to near-future improvements in seasonal forecasts [27,28], while sub-seasonal forecasts are consistently being improved each year [29].

## 5. Conclusions

In certain areas of Europe, there is potential for longer lead-times to be incorporated into pre-existing HHAP in the European Region, whilst countries yet to develop a HHAP could incorporate such information at the design stage. However, our results indicate that a compromise will have to be reached between user needs and the capabilities of seasonal climate forecasts over Europe, to provide skillful mortality predictions in advance of imminent extreme temperature events.
